# Muscle function and omega-3 fatty acids in the prediction of lean body mass after breast cancer treatment

**DOI:** 10.1186/2193-1801-2-681

**Published:** 2013-12-19

**Authors:** Cameron McDonald, Judy Bauer, Sandra Capra, Mary Waterhouse

**Affiliations:** University of Queensland, Brisbane, 4059 Australia

**Keywords:** Breast cancer, Omega-3 fatty acids, Lean body mass, Fitness, Nutrition, Exercise

## Abstract

**Background:**

Decreased lean body mass (LBM) is common in breast cancer survivors yet currently there is a lack of information regarding the determinants of LBM after treatment, in particular, the effect of physical activity and dietary factors, such as long-chain omega-3 fatty acids (LCn-3) on LBM and LBM function. This cross-sectional study explored associations of LBM and function with LCn-3 intake, dietary intake, inflammation, quality of life (QOL) and physical fitness in breast cancer survivors to improve clinical considerations when addressing body composition change.

**Methods:**

Forty-nine women who had completed treatment (surgery, radiation and/or chemotherapy) were assessed for body composition (BODPOD), LCn-3 content of erythrocytes, C-reactive protein (CRP), QOL, dietary intake, objective physical activity, 1-min push-ups, 1-min sit-stand, sub-maximal treadmill (TM) test, and handgrip strength.

**Results:**

After adjustment for age, LBM was associated with push-ups (r = 0.343, p = 0.000), stage reached on treadmill (StageTM) (r = 0.302, 0.001), % time spent ≥ moderate activity (Mod + Vig) (r = 0.228, p = 0.024). No associations were seen between anthropometric values and any treatment, diagnostic and demographical variables. Body mass, push-ups and StageTM accounted for 76.4% of the variability in LBM (adjusted r-square: 0.764, p = 0.000). After adjustment docosahexanoic acid (DHA) was positively associated with push-ups (β=0.399, p = 0.001), eicosapentanoic acid (EPA) was negatively associated with squats (r = −0.268, p = 0.041), with no other significant interactions found between LCn-3 and physical activity for LBM or LBM function.

**Conclusion:**

This is the first investigation to report that a higher weight adjusted LBM is associated with higher estimated aerobic fitness and ability to perform push-ups in breast cancer survivors. Potential LCn-3 and physical activity interactions on LBM require further exploration.

## Introduction

Loss of lean body mass (LBM) and simultaneous gains in fat mass are amongst the most common side effects following treatment for breast cancer (Mcdonald et al. 2011). This pattern of body composition change is distressing for the survivors and it is related to higher levels of chronic inflammation (Mourtzakis & Bedbrook 2009), and a greater risk for metabolic syndrome (Healy et al. 2010) and its related diseases (Healy et al. 2010; Pierce et al. 2009). A growing literature has established LBM, and in particular skeletal muscle tissue, as an influential organ in hormonal, immune and metabolic function (Pedersen & Febbraio 2012). Lifestyle factors such as physical activity and nutrient intake can enhance LBM size (Irwin et al. 2009) and function, (Courneya et al. 2007; Schmitz et al. 2005) and have also been associated with improved survival (Ibrahim & Al-Homaidh 2010) and quality of life (Mcneely et al. 2006) after treatment for breast cancer. Taken together, LBM is becoming an important marker for women who have been diagnosed with breast cancer.

Findings from observational studies have indicated that chemotherapy has been associated with declines of LBM during and after treatment (Cheney et al. 1994; Demark-Wahnefried et al. 1997; Demark-Wahnefried et al. 2001; Gordon et al. 2011; Kutynec et al. 1999), however not all trials have reported LBM loss after chemotherapy (Campbell et al. 2007). In contrast, associations between higher LBM and aromatase inhibitor hormonal therapy have been reported in three different data sets (Francini et al. 2006; Montagnani et al. 2008; Van Londen et al. 2011). Modifiable variables such as dietary intake and physical activity have not been extensively explored with regard to LBM change in breast cancer populations. Some evidence exists for an association between decreased physical activity and increased adiposity (Irwin et al. 2005), while mixed results have been reported in relation to dietary intake and adiposity, (Sheean et al. 2012) however a deeper understanding of physical activity, dietary factors and LBM change are needed to better guide clinicians in the post-treatment period.

Long chain omega-3 fatty acids (LCn-3) are established as anti-inflammatory agents and have been shown to protect LBM in cancer populations (Dewey et al. 2001; Murphy et al. 2012; Ries et al. 2012; Van Der Meij et al. 2011). However, conclusions from reviews of intervention studies in cancer populations investigating the effect of LCn-3’s on LBM have been mixed (Murphy et al. 2012; Ries et al. 2012). Typically, older studies have shown a protective effect for LBM when the appropriate dose of LCn-3 is consumed (Fearon et al. 2006; Fearon et al. 2003). More recent studies investigating 2 g of EPA LCn-3 supplementation in individuals undergoing chemotherapy for non-small cell lung cancer (NSCLC) have shown significantly greater attenuation of LBM and improved levels of intra-muscular triglyceride (IMTG), compared to those not supplementing. (Murphy et al. 2010; Murphy et al. 2011). In non-cancer populations the effect of LCn-3 on LBM has been minimal, with the majority of controlled trials indicating limited clinical effect (Mcdonald et al. 2013b).

Recent research has indicated that a greater effect may be seen when LCn-3 s are combined with an anabolic stimulus (Mcdonald et al. 2013b; Rodacki et al. 2012; Smith et al. 2011a; Smith et al. 2011b). Three small, well controlled studies combined LCn-3 supplementation with exposure to an anabolic stimulus, i.e. hyperinsulinaemic/hyperaminoacidaemic clamp or resistance training. Two reported an increased muscle protein synthetic (MPS) response to for young healthy (Smith et al. 2011b), and elderly participants (Smith et al. 2011a), yet LCn-3 alone made no difference to basal MPS. The third study that used resistance training reported increased peak torque development for the supplemented group above that of the group who received the resistance training program only (Rodacki et al. 2012). Considering LBM function, measured by strength or power development, may be more important to health outcomes than absolute values of LBM, (Newman et al. 2006; Ruiz et al. 2008) further investigations are required.

Therefore, the objectives of this cross-sectional study was to explore associations of LBM and LBM function in the context of LCn-3 intake, dietary energy and protein intake, inflammation, quality of life (QOL) and parameters of physical fitness and activity in women who had completed breast cancer treatment. A secondary goal was to determine the effect of interactions between tissue content of LCn-3 and markers of physical fitness on LBM after treatment for breast cancer.

## Methods

### Study design

All participants provided written informed consent. The data presented here was collected as the baseline assessment for a 6-month 3-arm randomized controlled trial (RCT) investigating LBM in women who have completed treatment for breast cancer. Detailed rationale study protocol for the full trial has been published previously (Mcdonald et al. 2013a). The study was approved by the Uniting Care (UCH HREC: #1034) and the University of Queensland (#2011000079).

### Participants

Participants were invited to participate through hospital breast cancer oncology centres, radio advertising, social media and breast cancer research registries in Brisbane, Australia. Baseline assessment occurred over one week, which included two visits 7 days apart.

### Eligibility

Women ≥18 years of age; had been diagnosed with early stage breast cancer (Stage 0-IIIa as determined by the American Joint Committee on Cancer Care); had successfully completed surgery, radiation and/or chemotherapy in the last 12 months (participants could be currently receiving endocrine and/or herceptin therapy); were able to perform moderate intensity physical activity, and have a BMI of >20 and <35 kg/m^2^ were eligible for enrolment. Participants were excluded if they had presence of metastatic growth or local/distal recurrence of cancer; a diagnosis of cardiovascular disease or diabetes; or, consumed >1 g of eicosapentanoic acid (EPA) and docosahexanoic acid (DHA) LCn-3 s combined per day.

## Measures

### Anthropometric variables

Height was measured to the nearest 0.5 cm using a stadiometer (Seca). Weight to the nearest 0.1 kg, LBM and fat mass were measured using the BODPOD digital scales and air displacement plethysmography (ADP) pod (BODPOD, COSMED USA Inc), respectively. Before each assessment day, the BODPOD scales and air chamber were calibrated as per the manufacturer’s instructions using known weights and volumes. All measures were performed by a certified BODPOD assessor. Results were expressed as percentage LBM and body fat of total weight, then absolute LBM was calculated giving a value in kilograms of LBM.

### Quality of Life (QOL)

QOL was measured using the Functional Assessment of Cancer Therapy- Breast + 4 (FACT-B + 4) tool (Cella et al. 1993). That FACT-F subset of questions was also added to capture participant fatigue. Higher scores are representative of better well-being.

### Diet history

Dietary intake was measured by the practitioner assisted Diet History Questionnaire (Martin 2004). Participants were asked to complete the questionnaire based on their intake over the last month. An accredited practicing dietitian reviewed the questionnaire with the participant to clarify portion sizes and other relevant details. Nutrient analysis was carried out using Foodworks 7 (Xyris Software).

### Blood analyses

Fasting high sensitivity-C Reactive Protein (CRP) was measured using a latex-enhanced immunoturbidimetric assay of blood serum. The 8.5 ml sample of whole blood was collected and analysed for CRP, then frozen at −20°C for transport to Victoria, Australia for fatty acid testing.

Lipids from red cells were extracted with chloroform methanol mixture. The fatty acids were trans-esterificated to methyl esters with methylation reagent “Meth-Prep 2”. The methylation extract was then analysed by gas liquid chromatography method with flame ionisation detection (gas chromatograph Schimadzu G-2010-FID). The proportion of fatty acids content of the erythrocytes expressed as % of total fatty acids.

### Muscle function and fitness tests

Grip strength was performed on both arms, with the maximum of 3 attempts recorded. Participants were seated with feet flat on floor, shoulder in neutral position with elbow bent at 90 degrees. Upper body muscular strength-endurance was measured using a 1-minute push-up test. Participants were asked to perform as many push-ups (knees on ground) as possible in 1 minute (American College of Sports Medicine 2010).

Lower body muscular endurance was measured using a 1-minute sit-stand test. The participant was asked to perform as many sit-stand movements as possible in 1 minute. Chair height was standardised at 43 cm height (American College of Sports Medicine 2010).

Sub-maximal aerobic capacity was measured using the modified Balke sub-maximal treadmill test. Seated blood pressure was measured before each assessment to ensure safety of exercise (Sharman & Stowasserb 2009). The test being completed when the individual had reached 85% of their estimated maximum heart rate (max HR) (est. maxHR = 220-age).

### Statistical analysis

Baseline characteristics were compared between treatment types and stages of disease using independent samples t tests or ANOVA. Spearman’s correlation coefficient was used assess the strength of bivariate associations, % time in moderate and vigorous activity were grouped together into one variable: % time in ≥ moderate activity. To assess the significance of age- and/or weight-adjusted associations between an outcome and a potential predictor, multivariable linear regression was used. Multivariable linear regression was used to model LBM as a function of various markers of fitness while also controlling for total body mass. For missing data, only those with full data sets were included in the models. The variables considered for inclusion in the model were those that were individually associated with LBM after adjusting for age and weight. Markers of fitness were added to the model sequentially, with the order determined by decreasing r-values. A predictor was only retained in the model if its coefficient was significantly different from zero at the 0.05 level. Adjusted R-squared was used to compare nested models. Models were also fitted that included interaction terms that explored the respective LCn-3 indices combined with fitness markers on LBM.

## Results

Participants were recruited over a 15-month period (Oct 2011 – Jan 2013). A total of 135 women were initially screened for inclusion criteria. The major reasons for exclusion were >12 months post treatment completion and daily consumption of >1000 mg EPA and DHA combined. Forty-nine participants were eligible for the study and completed baseline assessment. Descriptive statistics of the population are shown in Table 1.

**Table 1:** **Characteristics of participants**

Characteristic (n = 49)	Value
Age in years (mean; SD)	48.6 ± 9.5
Race (n, %)	
—Caucasian	44(88)
—Asian	3(6)
—African	1(2)
—Asian Pacific Islander	1(2)
**Anthropometric**	**(mean; SD)**
Height (m)	1.65 ± 0.07
BMI (kgm-2)	26.6 ± 4
Body mass (kg)	73.1 ± 13.8
LBM (kg)	43.6 ± 5.6
Body fat %	39.5 ± 6.9
Waist (cm)	85.4 ± 11.1
Hip Girth (cm)	106.3 ± 9.2
CRP (n = 45; med; range)*	(0.1–10.1)
Total % RBC n-3 (n = 43)*	5.9 ± 1.6
% EPA	1.1 ± 0.5
% DHA	2.9 ± 0.9
**Charaterstic of Disease (n; %)**	
0-I	13 (26)
IIa	19 (28)
IIb-IIIa	17 (34)
Estrogen receptor + ve	39 (78)
HER-2 receptor + ve	12 (24)
**Treatment variables (n; %)**	
Had Chemotherapy	41 (92)
Taxane – Yes	37 (74)
Radiation therapy	
Tamoxifen	13 (26)
AI	20 (40)
None	16 (32)
Time since completion Rx	165 ± 107

AI–Aromatase inhibitors.

*Missing data.

### Age

Age was positively correlated with improved breast cancer related QOL (r = 0.379, p = 0.007), fatigue (r = 0.311, p = 0.30) and EPA (r = 0.339, p = 0.026), and negatively correlated with % of time in vigorous activity (r = −0.342, p = 0.022) and number of squats performed in 1-minute (r = −0.363, p = 0.011).

### Associations of diagnostic and treatment related variables

Compared to those diagnosed with earlier stage disease (0-IIa), those with later stage disease (IIb-IIIa) reported poorer results for BrCa related QOL (89.2 ±9.3 vs 79.3 ±15.7; p = 0.009), fatigue (130.5 ± 15.3 vs 113.3 ± 24.7; p = 0.006), total score for Greene climacteric scale (11.8 ± 6.8 vs. 17.5 ± 10.2; F = 5.308, p = 0.026), with specifically worse symptom scores reported for psychological, anxiety, depression and somatic fields (all p < 0.05). Stage of disease was not associated with any indices of body composition, LCn-3 or physical function.

Compared to those who did not have radiation therapy, DHA values (t = 2.904; p = 0.016) and LCn-3: LCn-6 (t = 3.06; p = 0.004) ratios were higher for those who underwent radiation therapy. Otherwise, radiation therapy was not associated with markers of body composition, QOL, dietary intake, LBM function, endurance or physical activity. Individuals taking tamoxifen tended to have lower EPA content compared to those taking AIs or no hormonal therapy (0.78% vs. 1.16% & 1.23%; F = 3.153, p = 0.054), however, there was no evidence to support an association between hormonal treatment and other markers of body composition, QOL, dietary intake, LBM function or physical activity.

### Associations between LBM and dietary intake, inflammation, physical activity, markers of fitness and quality of life

LBM was positively correlated with daily intake of total energy (r = 0.301, p = 0.036) and protein (r = 0.464, p = 0.001), and negatively correlated with higher squat test results (r = −0.39, p = 0.006) (Table 2). However, after adjusting for weight and age, the only significant associations with LBM were % time spent in ≥ moderate intensity activity (ß: 0.228, p = 0.024), number of push-ups performed (ß: 0.343, p = 0.000) and treadmill stage completed (ß: 0.302, p = 0.001) (Table 2). CRP was positively correlated with body fat %, waist and hip however, these associations were no longer significant after controlling for total body weight (data not shown).

**Table 2:** **Associations between markers of absolute LBM and markers of LCn‒3 intake, dietary intake, physical activity and fitness adjusted for weight & age n value**

	Lean body mass (kg)				Body fat %			
	**Unadjusted**		**Adjusted**		**Unadjusted**		**Adjusted**	
	R	p-value	Standardise d B-coefficient	p-value	R	p-value	Standardise d B-coefficient	p-value
Total daily kJ intake	**0.301**	***0.036***	0.135	*0.132*	−0.82	*0.576*	−0.192	*0.065*
Total daily protein								
intake	**0.464**	***0.001***	0.144	*0.121*	0.04	0.787	−0.197	*0.068*
CRP*	0.258	*0.083*	−0.128	*0.238*	0.597	*0*	0.123	*0.343*
% time in sedentary								
activity*	0.167	*0.273*	0.053	*0.579*	0.23	*0.88*	−0.058	*−0.524*
% time in light activity*	−0.149	*0.329*	−0.104	*0.272*	0.156	*0.305*	0.125	*0.258*
% time in > moderate								
activity*	−0.041	*0.787*	**0.228**	***0.024***	−0.466	*0.001*	**−0.275**	***0.015***
Push up (in 1-min)*	−0.045	*0.760*	0.343	***0.000***	−0.671	*0*	**−0.457**	***0***
Squats (in 1-min)*	**−0.39**	***0.006***	0.044	*0.71*	−0.454	*0.001*	−0.098	*0.439*
Stage of treadmill								
completed	−0.047	*0.746*	**0.302**	***0.001***	−0.575	*0*	**−0.39**	***0***
FACT-B + 4	−0.13	*0.373*	0.038	*0.699*	−0.146	*0.316*	−0.009	*0.936*
FACT-F	−0.128	*0.381*	0.023	*0.813*	−0.133	*0.362*	−0.002	*0.982*

% time in activity: Accelerometry; % time in > moderate activity: moderate and vigorous activity grouped together; Stage of treadmill completed: at which 85% of estimated HRmax was reached; *Reduced data: CRP: n = 46; Accelerometer data: n = 45; Push-ups: n = 48; Squats: n = 48.

### Associations between LCn-3 and anthropometric indices, inflammation & quality of life after breast cancer treatment

No significant correlations were identified between absolute LBM or % LBM for total RBC n-3, ratio of AA: EPA or % RBC content of EPA or DHA (Table 3). No significant relationships were found between any other anthropometric variables and n-3 related values.

**Table 3:** **Univariate associations between indices of erththrocyte LCn-3 s and markers of body composition, inflammation and quality of life (n = 43)**

	Total n‒3		EPA		DHA		AA/EPA	
	r	*p-value*	r	*p-value*	r	*p-value*	r	*p-value*
Weight (kg)	0.083	*0.595*	0.249	*0.107*	−0.088	*0.576*	−0.232	0.134
LBM (kg)	0.093	*0.554*	0.156	*0.319*	−0.27	0.864	−0.159	*0.309*
Body fat %	0.037	*0.816*	0.222	*0.153*	−0.107	*0.493*	−0.181	*0.245*
Waist (cm)	0.123	*0.431*	0.280	*0.069*	−0.061	*0.697*	−0.167	*0.284*
Hip (cm)	0.055	*0.728*	0.280	*0.069*	−0.141	*0.366*	**−0.313**	***0.041***
CRP (mmol/L)	0.035	*0.822*	0.183	*0.241*	−0.42	*0.791*	−0.224	*0.149*
FACT-B + 4	−0.063	*0.689*	0.039	*0.804*	−0.064	*0.683*	−0.007	*0.962*
FACT-F	−0.129	*0.411*	0.040	*0.797*	−0.137	*0.382*	−0.026	*0.868*

LBM: Lean Body mass; CRP: C-reactive protein; EPA: eicosapentanoic acid; DHA: docosahexanoic acid; FACT-B + 4: Quality of life with breast related items; FACT-F: Fatigue.

No significant correlations were identified between CRP and erythrocyte LCn-3. No markers of body composition, CRP or indices of LCn-3 intake were significantly correlated with either measure of QOL.

#### Predictors of LBM in women soon after breast cancer

Number of push-ups, StageTM, and mod + vig activity were considered for inclusion in a weight-adjusted linear regression model for LBM (Table 2). Table 4 shows coefficients for the variables included in the final model. Table 4 also shows the value of adjusted R-squared obtained as each variable was successively added to the model. Mod + vig was not retained in the final model because the coefficient was not significantly different from zero (β=0.115, p = 0.177) in the presence of the other predictors. The model including weight, push ups and StageTM explained 76.4% of the variation in absolute LBM (Table 4).

**Table 4:** **Best predictors of LBM post‒treatment using hierarchical regression**

Predictor	Regression coefficient* (95% CI)	p-value**	Adjusted R^2t^
**Weight**	0.948	0.000	0.634
**Stage Tmill completed**	0.225	0.007	0.713
**No. push ups (1 min)**	0.275	0.002	0.764

*Regression coefficients taken from final model including: body mass, number push‒ups performed, treadmill stage reached.

**Significance of the individual predictor within the final model.

^t^Denotes value reported as each variable was added into the model in the order: body mass, stage tmill completed then no. push ups CI = confidence interval.

### Interactions of physical activity and indices of LCn-3 intake on markers of LBM function

The number of push-ups performed was positively correlated with time spent in ≥ moderate intensity activity (r = 0.467; p = 0.001), total n-3 levels (r = 0.385; p = 0.012) and DHA levels (r = 0.517, p = 0.000) (Table 5). The correlation with total n-3 levels was no longer statistically significant after adjusting for DHA. DHA maintained a significant association after adjusting for age, weight, LBM and % time > mod activity (β=0.399, p = 0.001) % ≥. Mod activity remained a significant predictor (F-Test: 8.95, p = 0.005) of the number of push-ups performed in one minute after adjusting for DHA, age, weight and LBM. There were no significant interactions between RBC LCn-3 and time spent in any intensity of activity for any of the regression models of physical function (data not shown).

**Table 5:** **Correlations between measures of physical function and LCn-3 content of erythrocytes**

Physical function*	% time>mod		EPA		EPA adj**		DHA		DHA adj.**	
	r	p-value	r	p-value	β	p-value	r	pcs-value	β	p-value
Push ups	0.467	0.001	0.072	0.648	0.212	0.118	0.517	0.000	0.399	0.001
Squats	0.110	0.479	−0.338	0.029	-0.268	0.041	0.153	0.333	-0.37	0.776
Handgrip	-0.068	0.663	0.083	0.603	-0.144	0.340	0.109	0.492	0.099	0.482
Treadmill	0.224	0.138	-0.11	0.493	0.13	0.929	0.267	0.083	0.147	0.277

*Push ups: performed in one-minute; Squats: performed in one-minute; Stage of treadmill completed: at which 85% of estimated HRmax was reached.

% time > mod: moderate & vigorous data combined; EPA: eicosapentanoic acid; DHA: docosahexanoic acid.

**Fully adjusted model included: weight, age, % time > mod activity & LBM. Correlation coefficient (β)

## Discussion

This paper reports a positive relationship between LBM (adjusted for total weight) and physical function represented by the % time spent in ≥ moderate intensity physical activity, stage achieved on sub-maximal treadmill test and number of push-ups completed. To the authors’ knowledge, this is the first study to determine associations between physical function and body composition in women who have completed treatment for breast cancer.

Our results agree with previous cross-sectional and prospective cohort studies, which have shown that decreasing physical activity levels are associated with greater adverse body composition change,(Irwin et al. 2003; Irwin et al. 2005) while dietary measures(Demark-Wahnefried et al. 2001) have been less predictive of these changes. The findings relating to the influence of chemotherapy on LBM agree with two previous studies (Campbell et al. 2007; Winters-Stone et al. 2009) but are in contrast to five studies that have shown a greater decrease in LBM after chemotherapy (Cheney et al. 1994; Demark-Wahnefried et al. 1997; Demark-Wahnefried et al. 2001; Gordon et al. 2011; Kutynec et al. 1999). In addition, Prado et al. reported that individuals with chemotherapy toxicity had a greater risk of sarcopenia (Prado et al. 2009). Differences in our results may be due to the cross-sectional nature of the study. Previously published data sets indicating LBM change after chemotherapy and hormonal therapies were prospective in nature (Cheney et al. 1994; Demark-Wahnefried et al. 1997; Demark-Wahnefried et al. 2001; Gordon et al. 2011; Kutynec et al. 1999) and were able to see trends over time.

No associations were found between erythrocyte LCn-3 and markers of body composition. Recent studies in populations during and post-chemotherapy treatment have indicated a positive relationship between skeletal muscle mass and plasma phospholipid LCn-3 content (Murphy et al. 2010; Murphy et al. 2011), however these participants experienced significant and rapid muscle loss during treatment. After early stage breast cancer treatment, the rate and magnitude of muscle loss experienced is not typically as high as when compared to more advanced staged cancers (Mcdonald et al. 2011; Murphy et al. 2010). As a result, our results are comparable to metabolic/obese populations undergoing similar body composition change (Krebs et al. 2006; Noreen et al. 2010; Storlien et al. 2001).

Total body mass, push-ups performed in one-minute, and stage completed on treadmill remained in the final model accounting for 76% of the variation in LBM. These results are of interest as they indicate an association with physical function and healthier body composition. Specifically, the strength of association with number of push-ups/minute as opposed to squats may indicate the importance of whole body resistance training to maintain or achieve a higher LBM and lower fat mass. A decrease in sports/recreational exercise has been previously associated with an increase in adiposity however, LBM change was not reported (Irwin et al. 2005). It is possible that those who performed more push-ups due to an increase in relevant exercise training may also be more conscientious in regards to dietary intake, however no association was found in this study.

Both erythrocyte DHA and EPA content were associated with markers of physical function, surprisingly in positive and negative directions, respectively. DHA was strongly and independently associated with the ability to perform push-ups, while erythrocyte EPA content was negatively associated with squats performed. In addition, assessing predictive models for push-up performance, when%time ≥ moderate physical activity was added to the DHA model, a greater effect was seen. In contrast, EPA content remained significantly negatively associated with squats performed. Previous studies have indicated an increase in muscle protein synthesis (Smith et al. 2011a; Smith et al. 2011b) and peak torque development (Rodacki et al. 2012) after supplementation of LCn-3 s was combined with an anabolic stimulus. In advanced cancer populations, EPA LCn-3 supplementation (often in conjunction with a protein-rich supplement) has been associated with improvements in physical function (Moses et al. 2004) and strength (Fearon et al. 2006), while EPA and DHA LCn-3 + NSAIDs have been shown to improve handgrip strength (Cerchietti et al. 2007). Our results both agree and disagree with the previous literature base with no clear reason for the opposing directions for the associations between physical function, DHA and EPA. Further investigation into LCn-3 and physical activity interactions are required.

Our population compared favourably with larger cohorts for body composition, (Chlebowski et al. 2002; Irwin et al. 2005) education level, (Irwin et al. 2005) however the exclusion of those with a diagnosed chronic disease (T2DM or CVD) and those who could not participate in moderate physical activity, may have led to our participants being younger and more physically active than the general breast cancer population.

## Conclusion

This is the first study to report that higher weight adjusted LBM is associated with greater upper body strength-endurance and aerobic fitness in women after completion of treatment for breast cancer. Further research is required to elucidate LCn-3-exercise interactions.
